# Racial differences in the relationship between high-normal 25-hydroxy vitamin d and parathyroid hormone levels in early stage chronic kidney disease

**DOI:** 10.1590/2175-8239-JBN-2020-0138

**Published:** 2020-10-05

**Authors:** Marquita B. Winder, Darius L. Mason, Janani Rangaswami, Arif Asif, Tushar J. Vachharajani, Roy O. Mathew

**Affiliations:** 1 Columbia Veterans Affairs Health Care System, Columbia, SC, United States.; 2 Methodist Le Bonheur Healthcare, Memphis, TN, United States.; 3 Einstein Medical Center, Philadelphia, PA, United States.; 4 Jersey Shore University Medical Center, Hackensack-Meridian School of Medicine, Neptune, NJ, United States.; 5 Cleveland Clinic Lerner College of Medicine of Case Western Reserve University, Glickman Urological & Kidney Institute, Department of Nephrology & Hypertension, Cleveland, OH, United States.; 6 University of South Carolina, School of Medicine, Columbia, SC, United States.

**Keywords:** Parathyroid Hormone, Renal Insufficiency, Chronic, African Americans, Chronic Kidney Disease-Mineral and Bone Disorder, Hormônio da Paratireóide, Insuficiência Renal Crônica, Afro-Americanos, Distúrbio Mineral e Ósseo na Doença Renal Crônica

## Abstract

**Aim:**

Current guidelines do not address between-person variability in markers of bone and mineral metabolism across subgroups of patients, nor delineate treatment strategies based upon such factors.

**Methods:**

A cross sectional study was carried out to analyze data from 20,494 United States Veterans and verify the variability of Vitamin D (25(OH)D) and parathyroid hormone (PTH) levels across race and stage of chronic kidney disease.

**Results:**

PTH levels were higher in Black Americans (BA) than White Americans (WA) at all levels of 25(OH)D and across eGFR strata. There was a progressive decline in PTH levels from the lowest (25(OH)D < 20) to highest quartile (25(OH)D >=40) in both BA (134.4 v 90 pg/mL, respectively) and WA (112.5 v 71.62 pg/mL) (p<0.001 for all comparisons).

**Conclusion:**

In this analysis, higher than normal 25(OH)D levels were well tolerated and associated with lower parathyroid hormone values in both blacks and whites. Black Americans had higher PTH values at every level of eGFR and 25(OH)D levels suggesting a single PTH target is not appropriate.

## RESUMO

**Objetivo:**

as diretrizes atuais não abordam a variabilidade entre as pessoas nos marcadores do metabolismo ósseo e mineral em subgrupos de pacientes, nem traçam estratégias de tratamento com base em tais fatores.

**Métodos:**

realizamos um estudo transversal para analisar dados de 20.494 veteranos de guerra dos Estados Unidos e verificar a variabilidade nos níveis de vitamina D (25 (OH) D) e hormônio da paratireóide (PTH) entre a raça e o estágio da doença renal crônica.

**Resultados:**

os níveis de PTH foram maiores em americanos negros (AN) do que em americanos brancos (AB) em todos os níveis de 25 (OH) D e em todos os estratos de TFGe. Houve um declínio progressivo nos níveis de PTH do quartil mais baixo (25 (OH) D <20) para o quartil mais alto (25 (OH) D> = 40) em AN (134,4 v 90 pg/mL, respectivamente) e AB (112,5 v 71,62 pg/mL) (p <0,001 para todas as comparações).

**Conclusão:**

Nesta análise, níveis de 25 (OH) D acima do normal foram bem tolerados e associados a valores mais baixos do hormônio da paratireóide em negros e brancos. Os americanos negros tinham valores de PTH mais altos em todos os níveis de TFGe e 25 (OH) D, sugerindo que uma única meta de PTH não é apropriado.

## INTRODUCTION

Established treatments for secondary hyperparathyroidism (SHPT) in chronic kidney disease (CKD) include phosphate binders, calcimimetics, and vitamin D sterols^1^^-^ 3. Vitamin D (25(OH)D) sterols, activated and nutritional vitamin D, have been used for decades to lower elevated parathyroid hormone (PTH) levels. Current guidelines do not address between-person variability in markers of mineral metabolism across subgroups of patients nor delineate treatment strategies based upon such factors even though this has been recommended by experts^4^.

There is evidence of racial differences in markers of mineral metabolism. In an analysis of community dwelling adults (approximately 6% with eGFR < 60 mL/min/1.73m^2^), Powe et al. showed lower levels of vitamin D-binding protein and associated lower total 25(OH)D levels in blacks compared to whites^5^. Among these patients, blacks had slightly higher PTH values than whites (39 vs. 34 pg/mL, p<0.001). Additionally, bone mineral density was higher among blacks than whites at any level of 25(OH)D, questioning the targeting of a single 25(OH)D level for bone health among racial groups. However, 25(OH)D supplementation is also utilized for suppression of PTH in CKD. Assessment of variation of PTH response to 25(OH)D has suggested differences among racial groups as well^4^. A study of vitamin D levels and PTH from a National Health and Nutrition Examination Survey dataset has alluded that PTH may be maximally suppressed at lower 25(OH)D levels in blacks than in whites^6^. Wright et al. also found that reductions in PTH was diminished at 25(OH)D level of ~20 ng/mL in African Americans versus ~30 ng/mL in Caucasians^7^. Ennis et al. concluded PTH levels in blacks were significantly higher than whites in CKD stage 2 through 5 with only a moderate component explained by 25(OH)D levels^8^. Gutierrez et al. noted an increase in PTH levels among blacks independent of serum 25(OH)D levels^9^. Thus, our aim was to explore if supra-normal 25(OH)D leads to suppression of PTH at earlier stages of CKD (CKD G3a) as compared to later (CKD G3b) and the influence of race on this relationship. We hypothesized that supra-normal (>40 ng/mL) levels of 25(OH)D are associated with lower PTH concentration. The effect should be: a) more pronounced in CKD G3a as compared to 3b, independent of race, and b) at any level of 25(OH)D, Black Americans (BA) would have higher PTH concentrations than White Americans (WA).

## METHODS

### DESIGN AND DATA

This was a cross-sectional study utilizing data from the Veterans Administration (VA) Corporate Data Warehouse (CDW) accessed through the Veterans Administration Informatics and Computing Infrastructure (VINCI). All veterans with values for estimated glomerular filtration rate (eGFR), 25(OH)D, and parathyroid hormone (PTH) between March to August 2013 were eligible for inclusion. Patients with an eGFR between 30 and 60 mL/min/1.73m^2^ at the beginning of this time period were included. One of the aims of this analysis was to assess interactions between 25(OH)D levels and the effect of race on PTH levels. Given the limited representation of non-Black or White Americans within the VA system, only patients listed as White or Black or African American were analyzed. For this evaluation, veterans with CKD stage 4 or greater, non-Caucasian and non-African American or unclear ethnicity, or primary hyperparathyroidism were excluded. Using the first 25(OH)D level within this time frame, we performed a cross-sectional correlation evaluation of 25(OH)D and contemporary (at the individual level) PTH values. Additional variables extracted included the presence of comorbid conditions including diabetes, hypertension, and seizure disorder. Medications of interest included cinacalcet, cholecalciferol, ergocalciferol, doxercalciferol, paricalcitol, medications with potential effects on vitamin D metabolism (rifampin, dilantin, carbamazepine), as well as prednisone. Patients on cinacalcet were excluded.

### STATISTICAL ANALYSIS

Baseline demographic and clinical variables were evaluated using descriptive statistics. Patients were divided by quartile of 25(OH)D level within the current cohort. Within each 25(OH)D quartile, patients were compared based on race (BA and WA). For descriptive comparisons, continuous variables were compared using parametric or non-parametric tests based on the underlying distribution; categorical variables were compared using Chi-square or Fisher’s exact tests based on the number of events in each cell of observation. Analysis of variance was utilized to compare PTH values across 25(OH)D levels in each racial category as well as by treatment with nutritional 25(OH)D. All statistical analyses were performed using R statistical software (version 3.5.1, R Foundation for Statistical Computing). The functions utilized for analysis included *glm* in the base package, *CreateTableOne* in the TableOne package, and *hist3d* in the plot3D package. Two-tailed p-value <0.05 was set as the limit for statistical significance.

## RESULTS

### GENERAL CHARACTERISTICS OF COHORT

A total of 5,065 BA and 15,429 WA were analyzed after inclusion and exclusion criteria were applied. Table 1 demonstrates the demographic characteristics at the time of evaluation, based on 25(OH)D level. There were fewer BA with 25(OH)D >40 ng/mL (16%) than WA (23%), and conversely more BA with lower (<20ng/mL) 25(OH)D levels than WA (30.4% vs 15.7%, respectively; p value <0.001). There was a significant increase in age as 25(OH)D levels increased in both BA and WA; at each quartile of 25(OH)D, WA were older than BA (p< 0.001).

**Table 1:** Demographic and clinical variables

	25(OH)D < 20		25(OH)D 20-29		25(OH)D 30-39		25(OH)D >=40		
	BA	WA	BA	WA	BA	WA	BA	WA	p
**N**	1542	2420	1567	4864	1151	4604	805	3541	
**Gender (males: N (%))**	1479 (95.9)	2327 (96.2)	1509 (96.3)	4686 (96.3)	1098 (95.4)	4428 (96.2)	743 (92.3)	3375 (95.3)	<0.001
**Age (years) (mean(sd))**	64.2 (11.1)	69 (10.2)	66.5 (11.1)	71 (10.3)	67.9 (11)	72.2 (10.2)	69.1 (10.6)	72.5 (10.3)	< 0.001
**Comorbidities (N(%))**									
**HTN**	806 (55.7)	1215 (53.0)	867 (57.2)	2552 (54.4)	651 (58.5)	2455 (55.1)	459 (59.1)	1860 (54.4)	0.01
**Diabetes Mellitus**	625 (43.2)	1087 (47.4)	659 (43.4)	2008 (42.8)	497 (44.7)	1723 (38.7)	297 (38.2)	1106 (32.3)	<0.001
**Osteoporosis**	67 (4.6)	120 (5.2)	82 (5.4)	324 (6.9)	58 (5.2)	426 (9.6)	55 (7.1)	427 (12.5)	<0.001
**Seizure disorder**	12 (0.8)	7 (0.3)	10 (0.7)	23 (0.5)	5 (0.4)	19 (0.4)	5 (0.6)	12 (0.4)	0.315
**Medications**									
**Steroids**	456 (29.8)	665 (27.9)	407 (26.2)	1174 (24.5)	331 (29.1)	1104 (24.4)	212 (26.6)	801 (23.2)	<0.001
**Antiretroviral Therapy**	46 (3.0)	7 (0.3)	29 (1.9)	21 (0.4)	20 (1.8)	21 (0.5)	12 (1.5)	20 (0.6)	<0.001
**Anti-epileptic drugs**	21 (1.4)	49 (2.1)	24 (1.5)	95 (2.0)	13 (1.1)	77 (1.7)	10 (1.3)	60 (1.7)	0.321
**Rifampin**	4 (0.3)	9 (0.4)	1 (0.1)	7 (0.1)	1 (0.1)	4 (0.1)	0 (0.0)	4 (0.1)	0.065
**Ergocalciferol 50,000 IU**	685 (44.8)	990 (41.6)	390 (25.1)	1063 (22.2)	171 (15.0)	441 (9.8)	158 (19.8)	329 (9.5)	<0.001
**Cholecalciferol 1000 IU**	576 (37.7)	888 (37.3)	667 (43.0)	1847 (38.6)	518 (45.6)	1411 (31.2)	331 (41.5)	968 (28.0)	<0.001
**Doxercalciferol**	2 (0.1)	2 (0.1)	2 (0.1)	7 (0.1)	0 (0.0)	5 (0.1)	2 (0.3)	6 (0.2)	0.832
**Paricalcitol**	3 (0.2)	2 (0.1)	2 (0.1)	3 (0.1)	1 (0.1)	7 (0.2)	0 (0.0)	4 (0.1)	0.779
**Vitamin D (400 IU)**	127 (8.3)	205 (8.6)	149 (9.6)	497 (10.4)	136 (12.0)	459 (10.2)	116 (14.6)	343 (9.9)	<0.001
**Calcitriol**	194 (12.7)	243 (10.2)	240 (15.5)	492 (10.3)	156 (13.7)	520 (11.5)	131 (16.4)	370 (10.7)	<0.001
**Laboratory Data**									
**PTH (mean (SD)) (pg/mL)**	134.35 (151.97)	112.52 (104.13)	111.81 (96.78)	91.55 (74.25)	100.93 (121.02)	79.89 (59.32)	90.02 (86.26)	71.62 (56.51)	<0.001
**25-hydroxy Vitamin D (mean (SD)) (ng/mL)**	14.11 (3.87)	15.01 (3.70)	24.92 (2.84)	25.10 (2.81)	34.33 (2.76)	34.52 (2.82)	50.68 (14.16)	50.16 (19.86)	<0.001
**Albumin (mean (SD)) (g/dL)**	3.67 (0.62)	3.70 (0.59)	3.80 (0.49)	3.85 (0.47)	3.86 (0.45)	3.91 (0.43)	3.90 (0.46)	3.92 (0.42)	<0.001
**Calcium (mean (SD)) (mg/dL)**	9.20 (0.81)	9.21 (0.91)	9.41 (0.73)	9.37 (0.73)	9.42 (0.63)	9.42 (0.70)	9.45 (0.61)	9.47 (0.72)	<0.001
**Glomerular Filtration Rate (mean (SD)) (mL/min/1.73m^2^)**	44.24 (8.29)	43.41 (8.26)	44.10 (8.67)	43.73 (8.32)	44.30 (8.51)	43.54 (8.23)	44.26 (8.24)	43.46 (8.20)	<0.001

BA: Black Americans; WA: White Americans.

BA had a higher proportion of hypertension at all quartiles of 25(OH)D (p<0.001). In the lowest quartile of 25(OH)D, diabetes was more common among WA than BA (47.4% vs 43.2%, p<0.001), and in the 3^rd^ quartile, diabetes was higher among BA than WA (44.7% vs 38.7%, p< 0.001). Osteoporosis was more common among WA than BA at all quartiles of 25(OH)D. Renal function was similar across groups, with BA having a marginally higher eGFR.

### MEDICATION USAGE

Vitamin D compounds: doxercalciferol and paricalcitol were not commonly prescribed. Ergocalciferol was more frequently prescribed at the lowest levels of 25(OH)D; at the highest quartile, BA were more likely to have a prescription of ergocalciferol than WA (19.8% vs 9.5%, respectively; p<0.001). Among BA, prescriptions for cholecalciferol, 400 IU vitamin D, and calcitriol were more common at higher 25(OH)D quartiles (p<0.001 for trend).

Medications that may affect vitamin D metabolism: steroids were more commonly prescribed among BA than WA (p<0.001 at all quartiles of 25(OH)D). Antiretroviral therapy use was low overall but significantly higher among BA than WA. Anti-epileptic drugs and rifampin use was low overall; differences between groups were non-significant.

### LABORATORY DATA: PRIMARY PTH ANALYSIS

**Figure 1:**
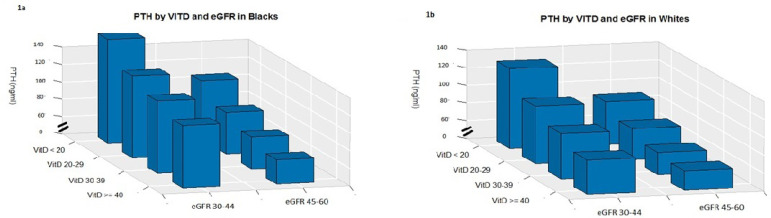
a. Parathyroid hormone (PTH) and vitamin D by estimated glomerular filtration rate in Black Americans; b. PTH and Vitamin D by eGFR in White Americans.

There was a progressive decline in PTH levels from the lowest (25(OH)D <20) to highest quartile (25(OH)D >=40) in both BA (134.4 vs 90 pg/mL, respectively) and WA (112.5 vs 71.62 pg/mL) (p<0.001 for all comparisons) (Table 1, Figures 1a, 1b). BA had higher PTH levels than WA at all levels of 25(OH)D. The effect of higher levels of 25(OH)D on PTH levels was greater at eGFR 45-59 as compared to 30-44 (Figures 1a, 1b).

ANOVA demonstrated significant effect of race (p<0.001) and vitamin D quartile (p<0.001), but not of the interaction between race and 25(OH)D quartile, suggesting that higher 25(OH)D levels are associated with lower PTH, independent of race despite BA having higher PTH levels than WA at every 25(OH)D quartile.

**Figure 2:**
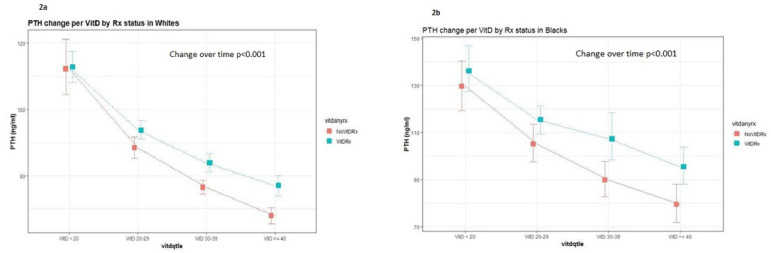
a. Parathyroid hormone (PTH) relationship to 25-OH vitamin D levels in White Americans with and without vitamin D supplementation; b. PTH relationship to 25-OH vitamin D levels in Black Americans with and without vitamin D supplementation.

Figures 2a and 2b graphically display the ANOVA listed above. Further, the graphs demonstrate that patients not on pharmacological vitamin D therapy have lower PTH levels at every 25(OH)D quartile.

### LABORATORY DATA: POTENTIAL ADVERSE EFFECTS

Higher levels of 25(OH)D were not associated with an increase in hypercalcemia (>10.5 mg/dL - corrected for serum albumin). OR (95% confidence interval) versus the lowest quartile of 25(OH)D were: 20-29 ng/mL, 0.94 (0.8-1.1); 30-39 ng/mL, 0.78 (0.65-1.49); >= 40 ng/mL, 0.81 (0.68-0.97)). Higher levels of 25(OH)D were also not associated with an increase in hyperphosphatemia (>3.4 mg/dL, the median for the cohort): OR (95% confidence interval) versus the lowest quartile of 25(OH)D: 20-29 ng/mL, 0.87 (0.79-0.95); 30-39 ng/mL, 0.89 (0.80-0.98); >= 40 ng/mL, 0.86 (0.77-0.95).

## DISCUSSION

The current analysis suggests that in CKD stage 3, higher than normal 25(OH)D levels are associated with lower PTH levels. This effect is similar among BA and WA. Higher than normal 25(OH)D levels were also not associated with hypercalcemia or hyperphosphatemia. In contrast, BA demonstrated higher PTH levels than WA at each category of 25(OH)D with or without vitamin D supplementation. Thus, nutritional vitamin D supplementation that raises 25(OH)D levels to higher than normal levels may be a safe and effective treatment of SHPT in early-stage CKD. Establishing an appropriate target level for PTH must take into consideration group differences that may be represented by features such as race.

In an effort to reverse or prevent the impact of hyperparathyroidism on the skeleton, vitamin D supplementation has been prescribed in patients with CKD for over three decades^10^. PTH has been the primary indicator for treatment adequacy in managing secondary hyperparathyroidism in CKD. Several studies have examined the ability to raise 25(OH)D levels to supra-normal (>40 ng/mL) levels and the influence of that increase on SHPTH parameters in patients with CKD. Sprague et al. examined two separate United States (US) cohorts undergoing identical clinical trials of calcifediol replacement in CKD stages 3 and 4^11^. Within these cohorts, calcifediol treatment resulted in a rise from mean value of 20 ng/mL at start of study to 70 ng/mL by week 25. This treatment also resulted in 30% reduction of PTH values in almost 60% of subjects by week 26, and at least 10% reduction in nearly 70% of participants. Few if any side effects were evident in this trial. Westerberg et al. demonstrated similar results in an analysis of a European cohort, with elevation of 25(OH)D levels from 23 to 65 ng/mL in 12 weeks with high dose cholecalciferol treatment^12^. There was a significant reduction in PTH levels, though the average reduction (~6.6 pg/ml) was modest. Likewise, these authors noted no side effect with these higher levels of 25(OH)D. The current analysis is consistent with these findings. In addition, the efficacy is evident, and seemingly more pronounced, at higher eGFR levels.

Westerberg and colleagues suggest a potential for high 25(OH)D target to prevent progression of SHPTH. A randomized controlled trial demonstrated that in 3,883 patients on hemodialysis and cinacalcet therapy, despite a significant reduction in PTH values, resulted in no statistically significant improvement in the primary cardiovascular endpoint^13^. In subgroup analysis, a shorter time on dialysis (<2 years) was associated with a trend toward benefit with cinacalcet therapy, though this did not meet significance. This suggests that treatment of SHPTH at the late stage of ESRD is inadequate for reversing or mitigating its effects on the cardiovascular health of patients. The appropriate stage of therapy is not clear. However, the current analysis as well as others^11^^,^ 14 ^,^ 15 suggest that effective control of SHPTH can be obtained with supra-normal levels of 25(OH)D at early CKD stage 3 levels. The current analysis further suggests that the control is more efficacious if started at the earliest stages of CKD. Long term studies are needed to assess whether this strategy will result in improvements in mortality and cardiovascular outcomes.

A growing body of evidence suggests that additional factors need to be considered including race-specific targets as there are differences in PTH when comparing Blacks versus Whites^16^^-^ 20. These differences have been reported across the continuum of CKD as well as in normal subjects^5^^,^ 21. Given our data on the differences in PTH values across race and different categories of 25(OH)D levels and eGFR values, if the optimization of SHPT is contingent upon targeting a particular range of PTH values, perhaps the target for PTH levels will need to be either race-specific or based on a threshold percentage reduction. However, unlike prior reports that have suggested a limited ability to reduce PTH by higher 25(OH)D levels^8^^,^ 9, the current analysis supports the use of supra-normal 25(OH)D for SHPTH in early stage CKD, independent of race. Again, prospective analyses are required to confirm this hypothesis.

There were limitations that need to be acknowledged in this analysis. First, this was a cross-sectional analysis of US veterans, who are primarily males. Discussions of SHPTH need to consider fibroblast growth factor-23 (FGF23) and klotho to form accurate assessments of links to important outcomes such as cardiovascular disease. These data were not available for the current analysis. Prior analyses have suggested no adverse effect of supra-normal 25(OH)D on FGF23 levels^12^. Prospective studies are needed to assess long-term effects of persistent supra-normal 25(OH)D levels on FGF23 levels in early stage CKD. Additional variables affecting bone health such as BMI and bone density were not available for this analysis.

In conclusion, the current analysis supports the finding, in a large cohort of US Veterans, that higher than normal 25(OH)D levels are associated with lower PTH levels in early-stage CKD. In addition, this analysis demonstrated that this effect is similar in BA and WA. However, the appropriate target for PTH is not clear as BA demonstrated a higher PTH at the same GFR or 25(OH)D level as WA. Thus, future treatment trials of SHPT in CKD, especially early stage CKD (3a and 3b), should consider high-dose vitamin D supplementation to lower or stabilize PTH levels, but will need to consider race, specifically in BA, when establishing PTH treatment targets.
